# Urological care for sexual and gender minorities: a narrative review toward equitable care

**DOI:** 10.1097/MOU.0000000000001393

**Published:** 2026-03-27

**Authors:** Hanna Hagen, Jojo Steininger

**Affiliations:** a Department of Urology, Klinik Favoriten, Vienna, Austria; b Department of Pediatric and Adolescent Medicine, Division of Pediatric Pulmonology, Allergology and Endocrinology; c Department of Social and Preventive Medicine, Center for Public Health, Medical University of Vienna, Vienna, Austria

**Keywords:** gender-affirming care, health inequities, sexual and gender minorities, transgender health, urology

## Abstract

**Purpose of review:**

Sexual and gender minority (SGM) populations face persistent inequities in urological care, stemming from stigma, biased frameworks, and inadequate clinician training. This review synthesizes current evidence on SGM-specific urological needs and highlights the importance of equity-oriented approaches to improve clinical outcomes and patient experiences.

**Recent findings:**

Across the urological care continuum, SGM populations experience lower cancer screening uptake, delayed diagnosis, poorer treatment-related quality of life, and unmet survivorship needs compared with cisgender and heterosexual populations. Traditional equality-based clinical guidelines frequently fail to account for anatomical diversity, gender-affirming hormone therapy, prior gender-affirming surgery, and psychosocial contexts that influence access to care and outcomes. In transgender and gender-diverse individuals, inconsistent application of organ-based screening, limited guidance following gender-affirming surgery, and under-recognition of long-term urological morbidity exacerbate inequities. Sexual and reproductive health needs, including trauma-informed care, fertility preservation, and inclusive approaches to sexual function, remain insufficiently integrated into routine urological practice. These disparities are reinforced by gaps in clinician knowledge and competence, limited collection of sexual orientation and gender identity data, and restrictive sociopolitical contexts.

**Summary:**

Equitable urological care for SGM populations requires moving beyond uniform, equality-based models toward anatomy-aware, affirming, and patient-centered care. Integrating equity principles into urological practice, education, research, and institutional policies is essential to improving outcomes and quality of care for SGM populations.

## INTRODUCTION

Structural inequities in healthcare profoundly shape the clinical experiences of sexual and gender minorities (SGM). The term SGM encompasses a diverse group of individuals whose sexual orientation, gender identity, gender expression, and/or sex characteristics differ from cisgender and heterosexual norms. This includes, amongst others, homosexual and bisexual individuals, transgender and gender diverse (TDG) people and individuals with differences in sex development (DSD). Despite substantial heterogeneity in identities and lived experiences, SGM populations share a common history of pathologization, stigmatization, and marginalization within medical systems – with enduring consequences for access to care, quality of care, and health outcomes [1].

Estimating the global size of SGM populations remains difficult due to a variability in definitions, data collection methods and sociopolitical contexts. National surveys in the global north indicate that up to 10% of adults identify as SGM, with increasing rates among younger generations [2–5]. It is estimated that 83% of SGM individuals worldwide conceal their sexual orientation from most or all people in their lives, reflecting the influence of country-level structural stigma on identity disclosure and visibility [6].

SGM individuals experience disproportionately negative interactions in medical settings and face substantial barriers to accessing care [1,7]. In clinical practice, inequities affecting SGM individuals are reinforced through institutional structures that reproduce cisheteronormative assumptions about bodies, identities, and sexuality; limited sensitivity among healthcare professionals; and persistent knowledge gaps regarding the lived experiences and healthcare needs of SGM individuals [1,8–13]. These challenges are particularly noticeable in urology, as many urologic conditions, diagnostic procedures and surgical interventions are closely linked to genital anatomy, sexual function, and reproductive health – domains in which SGM patients remain especially vulnerable to discrimination, misclassification, and inadequate or inappropriate care [8,14^▪▪^]. This contributes to delayed or avoided engagement with healthcare, diagnostic uncertainty, inappropriate management decisions, and poorer clinical outcomes for SGM patients [7,15–17]. Urological care frequently relies on equality-based assumptions that overlook the unique risks and needs SGM patients. This narrative review summarizes recent research on urological health in SGM populations, identifies key barriers to equitable care, and proposes strategies for advancing equity-focused practices. 

**Box 1:**
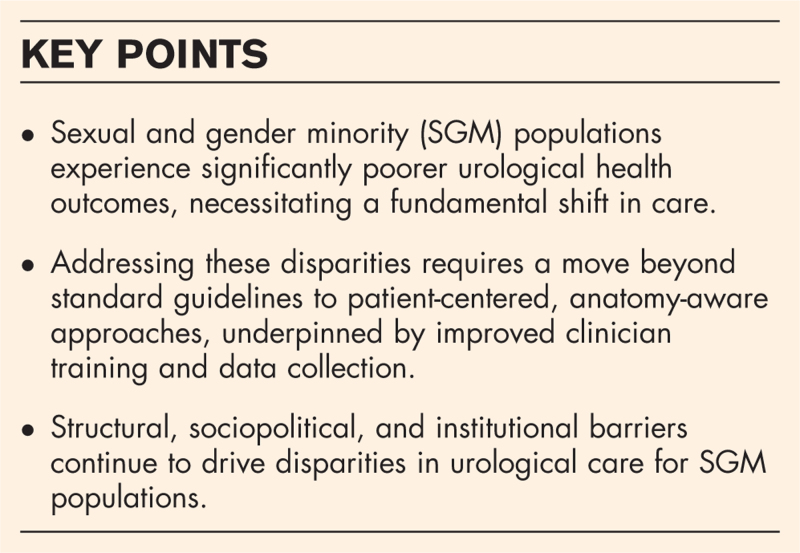
no caption available

## SEARCH METHODOLOGY

A literature review was performed using PubMed, Europe PMC, and Embase. A broad set of keywords related to urological healthcare for adult SGM was applied, with priority given to articles published within the last 18 months according to journal requirements. Consistent with a narrative review methodology, eligible publications included original research articles, observational studies, case reports and case series, as well as narrative and systematic reviews, restricted to English-language publications. Study selection was performed jointly by both authors.

## UROLOGICAL CARE FOR SEXUAL AND GENDER MINORITIES

This section summarizes recent evidence in urological care for SGM individuals, focusing on cancer, gender-affirming, and sexual/reproductive health. Despite increasing scientific attention, substantial disparities persist in delivering competent, evidence-based, and affirming care for SGM populations, including those with differences in sex development (DSD).

### Urological cancer care

Like other forms of cancer, urological malignancies disproportionately affect SGM populations, characterized by lower screening participation, diagnostic delays, and poorer outcomes compared to non-SGM individuals [15,18–22,23^▪▪^].

### Screening, risk assessment and diagnosis

Disparities in cancer screening highlight a critical entry point for inequity in urological cancer care for SGM populations. Current literature indicates that prostate cancer is relatively uncommon in transgender women [24]; however, emerging population-based data suggest that the risk among transgender and gender-diverse (TGD) individuals may be higher than previously assumed [25]. While gender-affirming hormone therapy (GAHT) substantially lowers prostate-specific antigen (PSA) levels, prostate cancer risk remains, suggesting underdetection rather than true risk reduction [24,26^▪▪^]. Consequently, GAHT may impact both prostate cancer risk and progression [24]. Current guidelines offer limited guidance on screening recommendations for TGD individuals, primarily utilizing a lower upper limit of normal PSA levels (1 ng/ml) [25,26^▪▪^,^27^,^28^]. Reduced PSA-screening uptake in TGD populations appears to be driven by the absence of clinician recommendation and counseling [29], further underscoring the need for proactive provider engagement.

Beyond prostate cancer, diagnostic inequities in urological malignancies – are largely shaped by structural, clinical, and psychosocial determinants of health. Although incidence of testicular cancer does not appear elevated among individuals receiving GAHT, diagnosis may be delayed due to avoidance of genital examination, limited anatomical familiarity and clinician discomfort [30–32]. Notably, recent case series reported higher-than-expected rates of germ cell neoplasms in gender-affirming orchiectomy specimens, highlighting gaps in surveillance and understanding [33].

In bladder cancer, SGM individuals experience poorer outcomes – likely related to increased risk factor burden (for example tobacco use), delayed evaluation, and limited provider familiarity with SGM-specific needs [23^▪▪^]. In TGD patients, prior gender-affirming surgery may necessitate modified cystoscopy or biopsy techniques, and failure to anticipate these differences can delay or compromise diagnosis [23^▪▪^,^30^].

### Cancer treatment and survivorship

Treatment outcomes for SGM patients with urological cancers are generally worse than of heterosexual and cisgender patients, particularly pertaining to survival, functional outcomes and quality of life [18,23,34–37^▪▪^]. Anatomy–aware approaches that account for prior gender–affirming care, sexual practices, organ preservation, and patient-centered quality–of–life goals are essential but often absent from standard oncologic care for SGM patients. In prostate and bladder cancer, altered anatomy resulting from prior surgery or hormone therapy may affect treatment tolerance, limit the feasibility of conventional techniques, and complicate future gender–affirming procedures [23^▪▪^,^30^,^38^,^39^].

Disparities persist in survivorship when counseling and follow–up fail to reflect diverse sexual practices, identities, and relationships. Safe identity disclosure, partner recognition, and access to SGM specific information are strongly associated with higher patient satisfaction across the cancer care continuum [38,40^▪^]. Sexual side effects, such as painful receptive intercourse, erectile or ejaculatory dysfunction, and changes in sexual roles, have distinct implications for SGM individuals after cancer treatment [41,42^▪^]. sexual minority men often report discrimination during cancer care, with substantial negative effects on health–related quality of life [41,43].

Although recent prostate cancer survivorship guidelines acknowledge SGM populations specific needs and recommend targeted sexual health interventions, evidence-based psychosocial programs tailored to SGM patients have not been developed yet [35,44]. Achieving equity therefore requires moving beyond uniform care models towards inclusive clinical frameworks that incorporate affirming communication and survivorship care centered on SGM patients’ lived experiences.

### Genital gender-affirming surgery

Genital gender–affirming surgery (GAS) is a cornerstone of care for many TGD individuals. Involving complex reconstruction of the lower urinary tract and pelvic floor, GAS positions urologists as key contributors across perioperative and long-term care [45]. Despite its clinical importance, access to GAS remains highly unequal due to limited surgical capacity, geographic and sociopolitical constraints, including limited insurance coverage and high out-of-pocket costs, resulting in long waiting times and significant distress [8,46–48].

Beyond access, GAS is associated with a substantial burden of urologic morbidity extending well beyond the immediate postoperative period. Common complications include voiding dysfunction, urethral complications, pelvic floor disorders, and persistent changes in urinary and sexual function [49^▪^,^50^,^51^]. Importantly, urinary and pelvic floor symptoms are influenced not only by surgical anatomy, but also by societal and structural factors – such as fear of discrimination and lack of safe, inclusive public spaces – which may further exacerbate lower urinary tract symptoms [49^▪^,^50^]. Emerging evidence suggests that postoperative morbidity may be systematically underestimated, with higher complication rates reported outside traditional surgical cohorts [51].

From an equity perspective, uniform expansion of surgical availability alone is insufficient. Advancing equity in gender-affirming urologic care requires preference-sensitive, identity-affirming counseling that reflects substantial heterogeneity in goals, priorities, and expectations [52]. In practice, this requires urologists to anticipate altered anatomy, adapt perioperative planning, provide individualized voiding and pelvic floor counseling, and ensure structured long-term follow-up.

### Sexual and reproductive health

Sexual health is central to urology, yet it is frequently under-addressed in clinical consultations, leading to under-treatment of sexual dysfunction and unmet patient needs [53–55]. These gaps are particularly pronounced among SGM populations [56–58]. Evidence-based guidance on sexual function and dysfunction in SGM individuals is limited by small, cross-sectional studies relying on cisgender, heteronormative models and lacking validated, inclusive outcome measures [59].

Beyond sexual function and satisfaction, sexual health inequities among SGM populations are evident in patterns of sexual risk, prevention, and harm. Elevated rates of sexually transmitted infections (STIs) in SGM populations are largely attributable to societal stigma and discrimination, barriers to STI testing, and gaps in sexual health education and risk awareness [9,60–64]. These challenges are further compounded by the high prevalence of prior sexual violence in SGM populations [65,66,67^▪^,^68^], underscoring the importance of trauma-informed approaches to sexual healthcare [69–71].

Normative assumptions about sexuality shape risk narratives and contribute to the marginalization of nondominant sexual identities, particularly asexuality. Persistent assumptions medicalize asexuality as dysfunction, rendering asexual individuals invisible within sexual health frameworks and mirroring parallel inequities in urologic care [72].

Similar normative assumptions also shape access to and experiences within reproductive healthcare, including fertility interventions [11,73]. Fertility preservation remains an important yet underutilized aspect of gender-affirming care, as medical interventions, especially GAS, can permanently affect fertility [74–78]. Current research is exploring testicular tissue cryopreservation and testicular sperm extraction as fertility preservation options for TGD youth. [75,79]. More research is needed to define success rates, optimize timing and evaluate long-term outcomes.

Collectively, this evidence highlights that achieving equity in sexual health requires care models that explicitly recognize and integrate the diverse sexual realities of SGM populations. For urologists, this necessitates routine, nonassumptive sexual history taking, trauma-informed communication, and proactive counseling on STI prevention, fertility preservation, and sexual function.

### Differences in sex development in adult urological care

DSD are underrepresented in urological care, creating unique clinical challenges for both patients and practitioners [80,81]. Historically managed in pediatric specialties, evolving guidelines question early elective surgeries, creating a growing demand for adult care providers. Case-based evidence highlights the need for lifelong follow-up, recognition of evolving gender identity, and individualized surgical planning, as early interventions may necessitate additional procedures later in life [82–84]. Equity challenges are particularly evident in surgical decision-making during childhood, where balancing malignancy risk, functional outcomes, fertility potential, and informed consent remains complex [80]. Shared decision-making frameworks and decision aids have emerged as important tools but are not yet widely implemented [85]. Adult urologists – especially those in reconstructive subspecialties – are more involved, despite persistent gaps in training and preparedness [86,87]. Ultimately, DSD care demands urological approaches prioritizing individualized, longitudinal care.

## BARRIERS TO EQUITABLE UROLOGICAL CARE

Intersecting structural, institutional, and sociopolitical barriers hinder equitable urological care for SGM populations. These inequities are compounded by stigma and persistent deficits in clinician education and research, resulting in compromised access, quality, and clinical outcomes.

### Provider knowledge, attitudes, and clinical competence

SGM individuals may encounter discriminatory comments or assumptions during routine clinical encounters, which are associated with poorer health-related quality of life [30,88,89]. Although many clinicians report feeling comfortable treating LGBTQ+ patients, sexual orientation and gender identity are frequently considered irrelevant to clinical assessment. This reinforces heteronormative assumptions, contributing to unmet sexual health and supportive care needs [56,90^▪▪^,91^▪^]. Empirical evidence demonstrates limited provider knowledge of sexual and gender diversity, persistent misconceptions, and widespread uncertainty regarding gender-affirming and SGM-inclusive clinical approaches [13,91^▪^,^92^,^93^]. These competencies directly translate into everyday clinical decisions – such as recommending organ-based cancer screening, confidently performing genital examinations, planning cystoscopy or biopsy after gender-affirming surgery, and providing timely counseling on fertility preservation and sexual function. Consequently, provider competence represents a central determinant of equity in urological care, influencing communication quality, patient trust, diagnostic accuracy, and engagement in care [90^▪▪^].

These competence gaps stem, in part, from structural deficiencies in clinical training. Qualitative studies reveal significant educational barriers for clinicians, including limited formal training, lack of mentorship, and restrictive sociopolitical environments [94,95]. Such environments may limit the inclusion of SGM health topics in medical curricula, constrain open discussion of gender-affirming care, and discourage LGBTQ-identifying trainees from pursuing relevant clinical expertise [94,96].

### Education and training

Deficits in formal education and training constitute a major provider-level barrier to equitable urologic care for SGM populations, reflecting systemic shortcomings in medical education rather than isolated individual factors [95,97–101]. Despite growing recognition of SGM health needs, significant gaps remain across medical education – from undergraduate training to continuing professional development. Limited clinical exposure to SGM patients and gender-affirming care contributes to clinician deficits in knowledge, confidence, and competence [102,103]. Learner demand for education in TGD care consistently exceeds current curricular offerings, underscoring a persistent mismatch between educational needs and training structures [103]. When SGM-related content is included, it is often fragmented, optional, or confined to theoretical instruction, with limited integration into clinical skills training, procedural education, or specialty-specific contexts [99–104].

Emerging evidence demonstrates that targeted, affirming educational interventions can improve clinician communication, confidence, and readiness to deliver equitable care. Systematic reviews show consistent improvements in attitudes, particularly when lived experiences are incorporated [105–107]. However, heterogeneity in training design and limited methodological quality highlight the need for standardized, competency-based, and outcome-oriented educational frameworks. Discipline-specific models show particular promise for advancing equity in urologic oncology and surgical care [108]. Without systematic integration into routine urological training, these gains remain inconsistent and dependent on individual motivation or experience. Embedding SGM-specific competencies within core urological curricula is therefore essential to ensure that equity-informed principles translate reliably into everyday clinical decision-making across screening, diagnostic evaluation, perioperative planning, and patient counseling.

### Data gaps

In most medical settings, collection of sexual orientation and gender identity (SOGI) data remains inconsistent, limiting the ability to characterize SGM–specific health outcomes and introducing bias into analyses of health disparities [109–112]. These substantial data gaps impede equity-oriented urologic care, particularly with respect to patient-centered outcomes [113–116]. Contributing factors include methodological limitations as well as participation avoidance due to noninclusive question framing, privacy concerns, and anticipated negative consequences when accessing care [117]. Without reliable SOGI data, clinicians and health systems lack the evidence needed to accurately assess risk, tailor screening strategies, evaluate treatment outcomes, and develop inclusive clinical guidelines, leaving urologic decision-making reliant on assumptions derived from cisgender and heterosexual populations. Addressing these limitations requires developing and implementing inclusive, validated outcome measures in urologic research and practice. Instruments like the GENDER-Q demonstrate the feasibility and necessity of patient-centered assessments – particularly for SGM populations – to achieve equitable care [118^▪▪^]. Furthermore, most existing research on SGM health originates from high-income countries in the global north, limiting generalizability and underscoring the need for more geographically and culturally diverse evidence bases, particularly in low- and middle-income countries (LMICs) [115].

### Sociopolitical barriers

Sociopolitical contexts strongly influence access to and quality of urologic and gender-affirming care for SGM individuals [95,119–121]. Laws, policy frameworks, and societal norms shape whether individuals seek medical care, disclose their sexual orientation or gender identity in clinical settings, and trust healthcare systems [122–125,126^▪^]. Restrictive legal environments, limited legal recognition, and negative societal attitudes can discourage care-seeking and promote concealment, while fear of discrimination often persists even in settings with formal legal protections [123].

Sociopolitical factors also affect access to care through insurance coverage, reimbursement policies, and eligibility criteria for urologic and gender-affirming services. Although inclusive policies and antidiscrimination protections can improve care utilization, their impact is reduced by inconsistent implementation, administrative complexity, and financial barriers [121,127]. Globally, these challenges are intensified by criminalization, socioeconomic marginalization, and limited healthcare infrastructure, particularly in low- and middle-income countries [128]. An intersectional perspective is essential, as sociopolitical barriers at the intersection of racism, homophobia, transphobia, and structural inequities disproportionately limit service access and worsen health disparities [129,130]. At the clinical level, these structural constraints translate into delayed presentations, interruptions in continuity of care, limited access to gender-affirming procedures, and reduced adherence to screening, treatment, and follow-up. Urologists may encounter patients who defer cancer screening, present with more advanced disease, lack insurance coverage for indicated procedures, or avoid postoperative follow-up due to prior negative healthcare experiences. These findings reveal systemic inequities in urologic care for SGM populations that extend beyond individual clinician behavior. Limited data, restrictive environments, and provider knowledge gaps cumulatively undermine equitable care and highlight the need for coordinated structural, educational, and policy-level responses.

## ACHIEVING EQUITABLE UROLOGICAL CARE FOR SEXUAL AND GENDER MINORITIES

Equitable urological care for SGM individuals must be tailored to patients’ specific anatomical, hormonal, and psychosocial contexts and supported by coordinated structural, educational, clinical, and research efforts.

### Institutional and structural strategies

Addressing inequities in urological care for SGM populations requires dismantling structural barriers through inclusive policies, nondiscriminatory practices, and improved access to gender-affirming and sexual healthcare. Creating safe, affirming clinic environments encourages engagement and appropriate care [131]. This includes practical measures such as inclusive intake forms, organ-based screening reminders, gender-neutral communication, and staff training in respectful examinations and procedures. Inclusive administrative practices, culturally competent staff, and nonassumptive intake processes further enhance patient trust and perceived quality of care [114,132].

However, policies and clinical guidelines alone are insufficient, as their impact is often limited by inconsistent uptake, resource constraints, and a lack of institutional accountability [101,104,109,133,134]. Implementation science frameworks can support the systematic integration of equity-oriented interventions into clinical workflows, training programs, and quality improvement initiatives [135].

### Clinician education and patient-centered care

High quality urological care also depends on clinician education and patient-centered approaches. Incorporating SGM-specific content into medical training improves preparedness, while patient-identified competencies – including professionalism, cultural competence, trauma-informed care, and shared decision-making – are critical to delivering affirming care [69,71,114,136]. Robust informed consent processes are equally essential. Standardized and accessible counseling tools, such as those addressing fertility preservation prior to gender-affirming interventions, support informed decision-making and may reduce decisional regret and psychological distress [137,138].

### Addressing the needs of aging sexual and gender minority populations

Urological care must anticipate the needs of aging SGM populations, who may face intersecting challenges related to comorbidity, social isolation, and the long-term consequences of earlier healthcare avoidance and exclusion [14^▪▪^]. Equity oriented care requires proactive planning, routine health maintenance, and targeted interventions to address these cumulative risks.

### Research and multidisciplinary care

Systematic inclusion of SOGI data in urological research is necessary to inform inclusive, evidence-based clinical guidelines. Achieving this requires institutional support and implementation strategies to ensure sustainability in routine care [132,139^▪^]. Multidisciplinary care models integrating urology, endocrinology, mental health, primary care, and community organizations further enable personalized and comprehensive gender-affirming care [140,141].

### Adapting frameworks to local legal, cultural and health-system conditions

Most of the available data and frameworks on SGM health originate from countries in the global north characterized by relatively accessible care and decriminalized minority status [142,143]. In contexts of legal and social marginalization or outright criminalization, SGM risk patterns and health access differs significantly [144–146]. Therefore, research frameworks must be adapted to local legal, cultural and health-system conditions. Increased participatory community-based research, especially research conducted in LMICs, is urgently required to inform urological care for SGM populations in highly stigmatized contexts [115,145]. Despite these limitations, the fundamentals of empathic care and affirming language should be applied by all urologists, regardless of legal settings or country-specific stigma.

Clinical implications for equitable urological care of sexual and gender minorities (Tables 1 and 2).

**Table 1:** Minimal standards of SGM-competent urological care

• Inclusive, nonassumptive language and safe opportunities for SOGI disclosure
• Organ-based (rather than gender-based) screening, diagnostics, and follow-up
• Documentation of prior gender-affirming treatments and their anatomical implications

**Table 2:** Urology-specific contexts requiring adapted care

• Cancer screening and diagnosis:
∘ Apply organ- and hormone-informed screening strategies
∘ Anticipate delayed presentation due to stigma or avoidance of genital examinations
• Procedures and diagnostics
∘ Plan cystoscopy, biopsy, and reconstructive procedures with anatomy-aware approaches
∘ Use trauma-informed communication prior to genital or invasive examinations
• Fertility preservation and reproductive health
∘ Address fertility implications before cancer treatment, gonadectomy, or gender-affirming surgery
• Postoperative care and survivorship
∘ Provide inclusive counseling on urinary, sexual, and quality-of-life outcomes

## CONCLUSION AND CALL TO ACTION

SGM populations experience persistent inequities across the spectrum of urological care, including urological cancer screening, treatment and survivorship, gender-affirming surgical interventions, and sexual and reproductive health. These disparities are driven by intersecting structural and sociopolitical barriers, gaps in clinician education and preparedness, and limited high-quality, inclusive evidence on SGM-specific urological needs.

Addressing these inequities requires a deliberate, field-wide commitment and coordinated action across institutions, healthcare systems, and national contexts. Key priorities include dismantling structural and sociopolitical barriers, implementing clear policies and accountability mechanisms to address stigma and discrimination, and improving clinician education to support patient-centered, anatomy- and trauma-informed care. Systematic and respectful collection of SOGI data is essential to inform evidence generation and guideline development, and clinical decision-making.

Future research must consistently center lived experiences and outcomes of SGM patients. Integrating qualitative and quantitative methods, as well as participatory research designs, will be critical to this effort.

Ultimately, equitable urological care for SGM populations must be understood as an integral component of high-quality, patient-centered urologic practice.

## Acknowledgements

*During the preparation of this work the authors used Perplexity AI (Perplexity, San Francisco, CA; Sonar model based on Llama 3.x) for spelling, grammar and language improvement only. After using this tool, the authors reviewed and edited the content as needed and take full responsibility for the content of the publication.*

### Financial support and sponsorship

*None.*

### Conflicts of interest

*There are no conflicts of interest.*

## REFERENCES AND RECOMMENDED READING

Papers of particular interest, published within the annual period of review, have been highlighted as:

▪ of special interest
▪▪ of outstanding interest
